# The Use of Steroid Injections for Hand and Wrist Pathologies

**DOI:** 10.7759/cureus.97104

**Published:** 2025-11-17

**Authors:** Rosemary J Bell, Rebecca Cervenak, Molly I Flint, Genevieve E Perrins, Amelia Dickinson, Aishwarya Ghosh, Shamim Umarji

**Affiliations:** 1 Medical Education, University of Queensland, Brisbane, AUS; 2 Internal Medicine, Princess Alexandra Hospital, Brisbane, AUS; 3 Internal Medicine, Royal Melbourne Hospital, Melbourne, AUS; 4 Internal Medicine, Grange University Hospital, Newport, GBR; 5 Palliative Care, Leeds Teaching Hospital Trust, Leeds, GBR; 6 Ophthalmology, Royal Victoria Infirmary, Newcastle Upon Tyne, GBR; 7 Trauma and Orthopaedics, St George's Hospital, London, GBR

**Keywords:** carpal tunnel syndrome, corticosteroid, de quervain's tenosynovitis, hand and wrist pain, intraarticular injection, osteoarthritis (oa), trigger finger disorder

## Abstract

Common hand pathologies that fail conservative management can be treated with corticosteroid injections. There are limited guidelines on the use of corticosteroids for hand and wrist pathologies. We aim to review the current practice of corticosteroid use and examine the available evidence regarding their efficacy and safety. A comprehensive literature review of PubMed and Cochrane databases was carried out from inception until present. Studies looking at the use of corticosteroids in common hand conditions in adults were included. Data on the dosage and timing of use of corticosteroids, side effects and complications were extracted. This evidence is summarised and compared with the current British and American guidelines. Corticosteroid injection into the wrist and hand are largely felt to be safe, with significant side effects uncommon. There are reported isolated cases of serious and irreversible complications such as necrotising fasciitis and flexor tendon rupture. The dosage of corticosteroid varied depending on the choice of steroid, but studies recommended a maximum of four injections a year, at a minimum three-month interval. The most appropriate choice of corticosteroids for all pathologies was betamethasone or methylprednisolone; however, this was largely based on weak evidence. Corticosteroids are used commonly for hand and wrist pathologies, but there is variable and conflicting evidence regarding the optimal dosage, timing, efficacy and safety profile of these medications. There is currently no evidence-based guidelines for treatment, with data being largely derived from larger joints. Further targeted research is needed to address this gap.

## Introduction and background

Hand pain is common [1], and underlying pathologies can cause significant disability [2]. Common pathologies include trigger finger (TF), carpal tunnel syndrome (CTS), De Quervain’s tenosynovitis (DQT) and osteoarthritis (OA). These pathologies, having failed conservative measures, can be well managed with corticosteroid injection alone or alongside other treatments [1].

Several steroids are currently available for injection, including triamcinolone, methylprednisolone, betamethasone and dexamethasone. All are associated with common side effects, including tendon rupture, infection, steroid flare, hypopigmentation, soft tissue atrophy, superficial radial nerve injury and hyperglycaemia in diabetic patients [3,4]. Current guidelines on the frequency and timing of corticosteroid injections, however, particularly in the hand and wrist, are limited, conflicting, and reflect a gap in the literature. There are also limited guidelines on corticosteroid injections for conditions in which the steroid is not injected intra-articularly, such as in TF and CTS.

The National Institute for Health and Care Excellence (NICE) recommends the use of intra-articular corticosteroid (IACS) injections to support patients to participate in therapeutic exercise [5]. The American College of Rheumatology [6] guidelines recommend IACS use in knee OA and suggest use in other sites of OA. The Osteoarthritis Research and French Society of Rheumatology guidelines also recommend IACS injections [6]. However, the American Academy of Orthopaedic Surgeons (AAOS) suggest that the balance between benefits and potential harms of corticosteroid injections was unclear [6]. The European Alliance of Associations for Rheumatology (EULAR) published guidelines in 2021 on intra-articular (IA) therapies, highlighting the scarcity of high-quality studies on the frequency and timing of IACS injections [7]. EULAR states that there are “no clear evidence-based recommendations as to the appropriate number of IA injections” with inconsistencies between studies [7]. EULAR recommends no more than three or four steroid injections into the same joint per year [7].

A comprehensive literature review was performed using the PubMed and Cochrane databases. We analysed studies relevant to our topic, focusing on the common hand conditions we have described which require corticosteroid injections. The British Society for Surgery of the Hand (BSSH) and the American Society for Surgery of the Hand (ASSH) guidelines [8-13] were searched for each of the outlined pathologies. The results were reviewed for relevant guidelines on the topic and the conditions described.

This paper aims to present an overview of our current understanding of steroid injections in the management of adults with TF, CTS, DQT and OA of the hand. We aim to investigate the optimal steroid to use for each condition, including its side effects, efficacy, the number of injections and the appropriate time interval between injections.

## Review

Common hand and wrist pathologies requiring steroid injection

Trigger Finger

Stenosing tenosynovitis is more commonly known as TF or trigger digit. It is commonly associated with stiffness and catching or locking, especially later in the course of the disease [8]. There is often a palpable and tender A1 pulley on examination [8].

Guidelines suggest non-operative management with splinting, education and steroid injection in simple first presentations [8,9]. Simple presentations here are those without previous surgery, locked digits or concurrent diabetes mellitus (DM) [9]. It is recommended that conservative methods are trialled for up to 12 weeks with a maximum of two injections per digit as per the BSSH [9] or three injections per digit per the ASSH [8]. There is a 60-90% chance of long-term resolution of symptoms with non-surgical treatment [8], but if symptoms are not adequately controlled, then a surgical approach is suggested [8,9].

Carpal Tunnel Syndrome

Median nerve compression in the hand is responsible for CTS [10]. It is the most common compressive neuropathy in the upper limb and frequently affects women between 45 and 60 years; pregnancy, hypothyroidism, OA of the wrist and DM are also risk factors [10]. CTS is characterised by pain and paraesthesia in the palmar aspect on the first three and a half fingers (median nerve distribution) and exacerbated by wrist flexion or pressure over the carpal tunnel [10]. The patient may develop loss of fine motor function and strength in the thumb [10].

First-line treatment for mild or moderate CTS is nocturnal splinting, activity modification, exercises and corticosteroid injections [10,11]. The BSSH suggests a single steroid injection and monitoring symptoms with non-operative measures for 12 weeks before moving to onward referrals and surgical consideration [11]. The ASSH also supports these recommendations but does not suggest a maximum number of injections [10]. If there are severe or red-flag symptoms, such as recurrent presentation, presentation following surgery or atypical symptoms, then a direct referral to a hand surgeon is recommended [11].

De Quervain’s Tenosynovitis

DQT is caused by inflammation of the tendon at the base of the thumb. It is characterised by swelling and pain over the radial side of the wrist, which is exacerbated by abduction, radial-ulnar deviation and palpation [8,12]. Recent childbirth and breast-feeding puts individuals at higher risk; whether this is due to repetitive movements of lifting the baby or hormonal changes is unknown [12].

The ASSH and BSSH suggest activity modulation, splinting and corticosteroid injections as non-operative treatment options [8,12]. Corticosteroids could relieve pain in up to 70% of cases [12]. The surgical alternative is a decompression of the compartments [8].

Osteoarthritis

OA is a chronic, degenerative process causing narrowing of the joint space, osteophytes and subchondral sclerosis [13]. Patients often present with progressive pain that is worse on movement [13]. Postmenopausal women and joints with repetitive load are at higher risk [13].

Both the ASSH and BSSH recommend a stepwise approach in OA of the thumb management [13,14]. First-line treatments include simple analgesia, activity modification, exercises and splints [13,14]. If non-invasive measures fail, IACS injections are recommended [13,14]. However, there is limited evidence that IACS injections are better than placebo [13]. The ASSH also suggests trialling hyaluronic acid (HA) injection, platelet-rich plasma (PRP) and/or autologous fat infiltration [13]. The next step following this would be surgical management [13,14].

Intra-articular corticosteroids used in practice

Triamcinolone

Triamcinolone acetonide (TA) and triamcinolone hexacetonide (TH) are commonly used in the management of musculoskeletal symptoms. Research has demonstrated that TH reduces the expression of citrullinated proteins, the monoclonal antibody F95 and peptidylarginine deiminase 4 in rheumatoid arthritis synovium, suggesting potential immunomodulatory effects [15].

Methylprednisolone

Methylprednisolone, a synthetic glucocorticoid, exerts its effects primarily through inhibition of phospholipase A2 (PA2), leading to a reduction in arachidonic acid derivatives such as prostaglandins and leukotrienes, which drive inflammation [16]. It also modulates immune cell activity by reducing leukocyte infiltration and suppressing pro-inflammatory cytokine production, including interleukins (ILs) and tumour necrosis factor alpha (TNF-α), thereby decreasing synovial inflammation and pain [17].

Dexamethasone

Dexamethasone is a synthetic glucocorticoid. It binds to glucocorticoid receptors, modulating gene expression to suppress pro-inflammatory cytokines, such as ILs and TNF-α, thereby reducing inflammation. Following administration, dexamethasone is absorbed into systemic circulation, with peak plasma concentrations varying based on the injection site [18].

Betamethasone

Betamethasone is a potent glucocorticoid that exerts its effects by binding to glucocorticoid receptors in the cytoplasm, forming a complex that translocates into the nucleus. This complex interacts with glucocorticoid response elements in DNA, leading to the upregulation of anti-inflammatory proteins, such as lipocortin-1, which inhibits PA2, and the downregulation of pro-inflammatory cytokines, including IL-1 and TNF-α [19,20]. This results in reduced synthesis of prostaglandins and leukotrienes, decreasing inflammation and immune response [21]. Additionally, betamethasone stabilises lysosomal membranes, reduces capillary permeability and suppresses leukocyte migration, further mitigating inflammation [22].

Efficacy

Multiple studies suggest that there may be limited long-term or functional benefit to utilising corticosteroids in the treatment of OA in the hand and wrist. While corticosteroids are known to provide short-term pain relief in carpometacarpal joint (CMCJ) OA, Kroon et al. concluded in their meta-analysis that IACS were no more effective than placebo in improving pain or function [23]. Furthermore, there was a lack of data on their use in interphalangeal OA, thus precluding drawing conclusions regarding efficacy in these joints [23].

Moreover, a systematic review conducted by Estee et al. showed no significant effect on clinical outcomes over short (four to six weeks) and longer (three to 12 months) terms, as well as no improvement in pain control or function in the short term [24]. A meta-analysis by Thakker et al. concluded that no corticosteroid injections, including IACS, were superior to placebo for short-term pain or functional improvements [25].  However, this conclusion was based on a small number of studies, and the authors recommend further research in this area. Dardas et al. performed a retrospective case study of 292 patients with TF requiring multiple injections. Despite symptomatic relief from multiple corticosteroid injections, 62% of participants ultimately required surgical release. The literature suggests that repeat corticosteroid injections are less effective than the initial injection [26]. 

Ayub et al. conducted a systematic review to assess the efficacy and safety profile of serial IACS injections for OA management. This review found no statistical difference in pain relief with repeated IACS injections in individuals with OA. However, frequent pain assessments in this trial were not undertaken, and the methods did not reflect clinical practice, as regular three-monthly injections were administered regardless of patient symptoms. Corticosteroid injections should typically only be repeated at individualised time intervals based on symptom control, with no more than four per year [27]. Donovan et al. investigated the effects of recurrent corticosteroid injections for knee OA and in trapezio-metacarpal joints between 3 and 24 months post-injection [28]. They found that recurrent corticosteroid injections were not beneficial in relieving symptoms, whereas injections with a placebo, HA and PRP were advantageous. However, trials analysed in this study had varying intervals, frequency and regimes for when these injections were given. They used 10 randomised controlled trials (RCTs), eight of which were focused on knee OA, and only two, both of which with small sample sizes, investigating trapezio-metacarpal OA. The lack of RCT data makes it applicability in the management of trapezio-metacarpal OA weak [28]. 

One study which challenged the lack of statistical significance was a 2015 systematic review by Fowler et al. [29]. This review suggested that there is a short-term, and possibly longer-term, benefit to using IACS in the trapezio-metacarpal joint. It reviewed nine RCTs and five prospective case series, concluding that IACS may offer significant short-term benefits, with only one study suggesting no benefit [29]. This review, however, only included studies with population sizes up to 83 participants and used variable methods, including case series with weak evidence and different steroid use, as well as concurrent treatment with other measures, such as splinting and analgesia, which could have influenced results. Studies published in languages other than English were excluded, potentially introducing bias. Furthermore, the study may be outdated, as it is based on research conducted before 2010 [29].

In TF management, it was found that 50% of a study population achieved symptom control within one or more years of recurrent steroid injections and that 39% of patients who received second and third corticosteroid injections achieved long-term symptom relief [26]. In CTS management, one corticosteroid injection was found to provide symptom relief for up to six months, and regular injections have been suggested as an alternative to surgical management [30]. A meta-analysis by Adindu et al. compared corticosteroid injections to placebo and wrist splints in CTS, reporting a statistically significant, but not clinically meaningful, improvement in symptoms with corticosteroid injections. Electrodiagnostic testing demonstrated improvement in motor and sensory function at three months following corticosteroids but not at six months [31].

Side effects and complications of corticosteroid injections

There are low rates of major adverse events associated with corticosteroid use in the wrist and hand, with 37.5% of studies reporting no side effects and no deaths [32-37]. The most reported side effects were skin depigmentation in 1.3-4% of cases [38-40], atrophy of the subcutaneous tissues in 1.5-40% of cases [38,39,41-43] and cellulitis in 4% of cases [44]. It should be noted that these effects are generally self-limiting with resolution within 9 to 12 months [45,46].

Although deep infection is extremely rare, there have been isolated cases reported: Yam et al. reported a case of necrotising fasciitis following corticosteroid injection for TF [47], and Baack and Brown reported a case of atypical mycobacterium infection following corticosteroid injection for DQT [48].

Despite corticosteroid injection being primarily localised in effect, systemic absorption can lead to temporary suppression of the hypothalamic-pituitary-adrenal (HPA) axis, limiting response to stress and infection. Cortisol levels typically recover within one to four weeks following Methylprednisolone injection and within 72 hours of Dexamethasone injection [49-51].

Tendon rupture is also listed as a major side effect. There is only one reported case after corticosteroid injection into the carpal tunnel for median nerve compression syndrome [52], resulting in bilateral digital flexor tendon rupture. The mechanism is thought to be secondary to corticosteroids reducing the tensile strength of the tendons, increasing the risk of rupture [53].

Vascular side effects have also been reported. One case of digital ischemia occurred after carpal tunnel injection, likely due to vasospasm, though it resulted in no permanent damage [54]. More concerning was a case of radial artery ischemia following corticosteroid injection into the wrist [55], thought to be due to atypical anatomy. Transient facial flushing is the most frequently reported side effect from TA and TH, commonly occurring two to three days post-injection. This reaction is generally mild [15].

IACS use is also linked with potential cartilage damage, such as reduced cartilage thickness, particularly with repeated or high dose injections [56]. In vitro studies have shown triamcinolone to be chondrotoxic at all tested doses, while in vivo studies suggest low doses of methylprednisolone, hydrocortisone and triamcinolone may be chondroprotective [57]. Variability in study findings can be attributed to differences in animal models, dosage regimens and treatment durations [57]. Dexamethasone may have protective effects due to its anti-inflammatory properties, though its long-term effects on cartilage remain uncertain [51,58]. In contrast, frequent or high-dose betamethasone disrupts cartilage homeostasis which may risk joint degeneration [59]. A pivotal RCT by McAlindon et al. found that 40 mg of TA every three months for two years led to greater cartilage loss without significant improvement in pain versus placebo [50]. Although cartilage loss does not worsen symptoms directly, it has been associated with higher rates of arthroplasty [51,60].

Up to 42% of IACS injections may be extra-articular when imaging is not utilised, and up to 25% of the compound may extravasate into surrounding soft tissues [40]. This could affect the efficacy and dosing of the drug; therefore, the use of fluoroscopy or alternative imaging may improve outcomes [40].

Corticosteroid injections into the wrist and hand, therefore, are largely thought to be safe and without serious side effects [61]. There are multiple well-researched side effects and complications of corticosteroid injections (Table 1). Reviews on this topic can divide side effects into common, less common and very uncommon or rare side effects, and these can be used to relay information to patients when consenting for procedures. The very uncommon or rare complications reported in literature include deep infection (<0.01%), tendon or pulley rupture (<0.1% for individual risk) and transient superficial nerve irritation. More commonly seen in 25% of patients is post-injection flare, and 5.8% of patients may experience soft tissue pain or cellulitis such as reactions. Skin changes such as hypopigmentation (1 in 3,000) or local atrophy (1 in 50,000) occur less commonly, while hyperglycaemia lasting up to five days is typical in diabetic patients, although infrequently requires medication adjustment [5,6,40].

**Table 1:** A summary of side effects and complications of corticosteroid injections

Complication	Incidence/Frequency	Key Studies	Clinical Notes/Implications
Deep infection	<0.01%; isolated case reports	[47,48]	Necrotising fasciitis and atypical mycobacterial infection reported, extremely rare but serious.
Tendon rupture	Single reported case	[52,53]	Bilateral digital flexor rupture after carpal tunnel injection, mechanism likely steroid-induced tendon weakening.
Skin hypopigmentation	1.3–4%	[38-40]	Typically self-limiting, cosmetic concern.
Subcutaneous atrophy	1.5–40%	[38,39,41-44]	Usually resolves within nine to 12 months.
Cellulitis	Up to 4%	[44]	Localised infection, responds to antibiotics.
Post-injection flare	Up to 25%	[15,40]	Transient pain/swelling, usually self-resolving.
Vascular events such as ischaemia	Rare (isolated reports)	[54,55]	Digital or radial artery ischaemia, typically reversible, consider anatomical variation and injection technique.
Facial flushing	Common; transient	[15]	Occurs two to three days post-injection, especially with triamcinolone; benign.
Hypothalamic-pituitary-adrenal axis suppression	Transient; duration three days to four weeks	[49–51]	No long-term harm expected in most patients; relevant in those with adrenal insufficiency.
Cartilage damage (osteoarthritis-related joints)	Possibly dose-dependent; cartilage loss in RCT	[50,57,59,60]	Risk with repeated/high-dose use; cartilage loss not always symptomatically evident but may affect joint outcomes.
Extra-articular injection	Up to 42% without imaging; 25% extravasation	[40]	May reduce efficacy and increase risk of local side effects; imaging guidance recommended.

Dosage, number and timing of steroid injections 

A widely accepted injection interval into the same joint is three to four months [7]. Guermazi et al. reported that injections are often administered up to four times per year, consistent with NICE guidelines [56]. Despite this, EULAR notes that there is no objective limit on the number of corticosteroid injections a patient should receive, with long-term safety of repeat injections not well reported [7]. Consequently, the decision to repeat injections is determined by a clinician’s judgement, patient response to previous injections and preference, rather than a strict evidence-based practice [62].

Peri-operative timing of injections is important with studies investigating hip and knee conditions, suggesting a gap of four weeks between steroid injection and the procedure to reduce the risk of postoperative infection [63]. For upper limb procedures, it is recommended to wait at least three months preoperatively and one month postoperatively [64]. Qin et al. reported a greater risk of surgical complication or infection in patients who received an IACS injection up to three months before thumb CMCJ surgery [65]. The recommended doses and timings for corticosteroid injections is summarised in Table 2.

**Table 2:** A summary of recommended dosages and timings for corticosteroid injections

Corticosteroid	Typical Dose (Small Joint)	Duration of Effect	Notes
Triamcinolone hexacetonide	20 mg	Two to three weeks	Least soluble; prolonged effect [66]
Triamcinolone acetonide	10–40 mg	Approximately 14 days	Commonly used for OA [66]
Methylprednisolone	4–10 mg (small joints)	One to five weeks	Rapid onset (within one week) [67]
Dexamethasone	0.8–1 mg	Up to 72 hours	Can be repeated every three days [68]
Betamethasone	0.25–0.5 mL (30 mg/5 mL)	One to two weeks	Three to four injections for tenosynovitis [69]

In summary, studies recommend spacing joint injections by at least three months, with a maximum of four injections per year per joint being considered within the window of clinical safety standards [7,56]. Repeat injections should, however, be an individualised approach, considering response and pathology balanced against risk of cumulative adverse effects and diminishing returns of therapeutic potential.

Choice of corticosteroid 

A survey conducted by the AAOS reported that over 90% of 233 orthopaedic surgeons routinely use corticosteroid injections. However, the absence of standardised clinical guidelines has led to considerable variability in practice and potential overuse of steroids beyond recommended dosages [70]. This variability was demonstrated in a study by Skedros et al. who reported that 29% of sports and exercise physicians, 41% of orthopaedic surgeons and 44% of rheumatologists exceeded the recommended steroid dose when injecting the acromioclavicular joint [71]. The selection of an appropriate steroid should be based on multiple factors, including the underlying condition, the intended duration of treatment, the therapeutic goal (symptomatic relief versus delaying surgery) and the stage of disease progression.

TA and methylprednisolone are generally considered the most effective options for wrist conditions due to their potent anti-inflammatory properties and relatively lower risk of adverse effects [4]. Injections of TA and TH are well-tolerated, with a low incidence of side effects comparable to other corticosteroids [15]. Betamethasone may provide longer-lasting relief in severe cases, but it is associated with a higher incidence of side effects [72]. In contrast, dexamethasone is often preferred for short-term relief in acute conditions but may be less effective for chronic disease management. A summary of the most appropriate corticosteroids for various hand and wrist pathologies is provided in Table 3.

**Table 3:** A summary of the most appropriate corticosteroids for various hand and wrist pathologies

Condition	Most Appropriate Steroid	Short-Term Relief	Long-Term Relief	Strength of Evidence	Key Points	
Carpal tunnel syndrome	1 mL of betamethasone sodium phosphate or 40 mg/mL of methylprednisolone (Depo-Medrol). 20 mg is noninferior to 40 mg.	Good	Weak	Weak	Provides significant short-term pain/function improvement. May reduce need for surgery, but up to 50% recurrence [73-75].	
de Quervain’s tenosynovitis	1 mL of betamethasone sodium phosphate or methylprednisolone.	Fair	Not available	Weak	Shows short-term pain relief, but long-term data are lacking [73-75].	
Trigger finger	0.5 mL to 1 mL of betamethasone sodium phosphate or methylprednisolone.	Fair to good	Fair	Fair	Cure rates range from 54% to 86%, making it one of the most effective conditions for corticosteroid injection use in both short- and long-term relief [73-75].	
Wrist and hand osteoarthritis	0.25 mL to 0.5 mL of betamethasone sodium phosphate or methylprednisolone. Studies used 10–20 mg of methylprednisolone/triamcinolone for small joints.	Not available	Weak	Weak	No strong recommendation from the American College of Rheumatology on corticosteroid injections for Osteoarthritis [73-75].	

Studies suggest that corticosteroids provide varying levels of short- and long-term relief depending on the condition, with CTS showing good short-term response but high recurrence rates, and TF demonstrating one of the highest success rates with cure rates between 54% and 86% [70,71]. While methylprednisolone and triamcinolone are commonly used, evidence indicates that TH may be superior for interphalangeal joint injections. Additionally, for wrist joints, 20 mg of methylprednisolone has been found to be noninferior to 40 mg, highlighting that higher doses may not always provide additional benefit [4]. However, no studies have specifically evaluated the impact of injection volume on clinical outcomes.

## Conclusions

Despite the widespread use of corticosteroids in practice, the evidence defining the optimal type, dosage, timing, efficacy and safety profile for hand and wrist pathologies is variable and conflicting. The data available are primarily derived from larger joints, particularly knee OA, and its applicability to smaller joints, such as the hand and the wrist, remain uncertain. Current research suggests that corticosteroids provide limited long-term improvement in symptoms for conditions such as OA of the base of the thumb, CTS and TF, but the best long-term response is observed in TF. Despite limited evidence, corticosteroids may still offer short-term relief. 

While generally considered a safe procedure, the long-term risk of serious side effects from corticosteroid injection requires further investigation. There is also a lack of clear guidance on the optimal timing and total lifetime dose of repeat corticosteroid injections. Further research is needed to establish specific recommendations for different sites, including different joints, and pathologies. Alternative treatments, such as HA and PRP, are being explored and may offer more effective options. Comprehensive research on steroid use in hand and wrist pathologies is essential to develop evidence-based guidelines for treatment.
